# Epidemiological Research on Hand, Foot, and Mouth Disease in Mainland China

**DOI:** 10.3390/v7122947

**Published:** 2015-12-07

**Authors:** Zhi-Chao Zhuang, Zeng-Qiang Kou, Yong-Juan Bai, Xiang Cong, Li-Hong Wang, Chun Li, Li Zhao, Xue-Jie Yu, Zhi-Yu Wang, Hong-Ling Wen

**Affiliations:** 1 Department of Virology, School of Public Health, Shandong University, Jinan 250012, China; dennis.eyre@hotmail.com (Z.-C.Z.); 201413969@mail.sdu.edu.cn (Y.-J.B.); 201514148@mail.sdu.edu.cn (L.-H.W.); 201514134@mail.sdu.edu.cn (C.L.); dlzhl@sdu.edu.cn (L.Z.); yuxuejie@sdu.edu.cn (X.-J.Y.); zhiyu.wang@sdu.edu.cn (Z.-Y.W.); 2 Shandong Center for Disease Control and Prevention, Jinan 250014, China; jack-cou@163.com; 3 Qilu Hospital of Shandong University, Jinan 250012, China; cxonway1107@163.com; 4 Center for Biodefense and Emerging Infectious Diseases, Departments of Pathology and Microbiology, Institute for Human Infections and Immunity, University of Texas Medical Branch, Galveston, TX 77555-0609, USA

**Keywords:** HFMD, enterovirus, epidemiology, etiology, influencing factor

## Abstract

Hand, foot, and mouth disease (HFMD), which has led to millions of attacks and several outbreaks across the world and become more predominant in Asia-Pacific Region, especially in Mainland China, is caused by several Human Enteroviruses including new enterovirus, coxsakievirus and echovirus. In recent years, much research has focused on the epidemiological characteristics of HFMD. In this article, multiple characteristics of HFMD such as basic epidemiology, etiology and molecular epidemiology; influencing factors; detection; and surveillance are reviewed, as these can be help protect high risks groups, prevalence prediction and policy making for disease prevention.

## 1. Introduction

Hand, foot, and mouth disease (HFMD) was first reported in 1957 in New Zealand. It was named after its general clinical characteristics, and has led to millions of attacks and several outbreaks across the world. In the past decade, HFMD has become more predominant in the Asia-Pacific Region [1,2,3,4,5,6,7] and has become a pressing problem for global public health. After large outbreak in 2008, the incidence of HFMD in Singapore has varied from 346.4/100,000 in 2009 to 698.8/100,000 in 2012. Coxsakievirus A16 (CVA16) and Coxsakievirus A6 (CVA6) were predominant in HFMD cases. Moreover, about half of the cases were co-infected with two or three pathogens [8]. In 2009, an outbreak of HFMD first occurred in the Republic of Korea [9], since then, outbreaks have been continually reported in Korea. In 2010, a large outbreak of HFMD occurred in Hong Kong, leading to more than triple of the annual average cases reported during the year 2006 to 2009 [10]. Besides, the number of cases in children older than five years continually increased from 2001 to 2009. Vietnam suffered from a large HFMD outbreak in 2011, leading to about 113,121 cases and 170 deaths [11]. In 2012, more than 40,000 suspected HFMD cases were reported during the rainy season in Thailand [12], and CVA6 played an important role in this large outbreak. After two consecutive years of outbreaks that caused hundreds of thousands of cases and thousands of deaths [4], the Chinese government classified HFMD as a C-class notifiable communicable disease in 2008. HFMD usually attacks children under the age of five [13,14], causing mild symptoms including fever, erythra, vesiculation, and inappetence, but some symptoms are extremely severe and are accompanied by several neurological complications such as encephalitis, cephalomeningitis and neurogenetic pneumonedema; circulatory disturbance; and can even lead to death. Human enteroviruses (new enterovirus, coxsakievirus and echovirus) are the most common pathogenic agents of HFMD. The major and most important causative agents are enterovirus 71 (EV71) and coxsakievirus A16 (CVA16) [14,15], since their infections can result in severe consequences [16], especially EV71, which can result in the most severe consequences. Furthermore, specimens initially considered to be infected with CVA16 were found to be co-infected with CVA16 and EV71 after inspection. Although the development of HFMD vaccines has rapidly expanded and has already finished pre-clinical studies and even completed several clinical phases [17,18,19,20], there are still no available vaccines and effective anti-virus therapies for HFMD. Epidemiological studies of HFMD can obtain a better understanding and provide potential possibilities for the control, prevention, and surveillance of HFMD.

## 2. Basic Epidemiology

In March 2008, a sudden outbreak of HFMD occurred in Fuyang, Anhui Province. Soon after, a number of cases spread across the country and by the middle of June, the caseload reached about 20,000, including dozens of deaths. On 2 May 2008, HFMD was defined as a C-class notifiable disease in Mainland China.

According to the HFMD surveillance system, the incidence of HFMD from 2009 to 2014 in Mainland China was 86.59/100,000, 132.35/100,000, 120.21/100,000, 160.17/100,000, 134.37/100,000, and 203.16/100,000, respectively, while the mortality was 30.55/100,000, 51.00/100,000, 31.42/100,000, 21.64/100,000, 13.78/100,000, and 18.03/100,000, respectively. The difference of incidence of HFMD among male and female cases was observed in several studies [14,15,21,22,23,24,25,26]. Xing *et al.* [2] found that the incidence of HFMD in males under the age of five was 1.6 times higher than that in females, and Deng *et al.* [21] found that about 65% of HFMD cases were boys, while the percentage was 62.4% for Zhang *et al.* [22]. Data collected by our research team in 2009, 2010, and 2014 showed that the prevalence of male was about 1.5 times that of females. However, so far, none of these studies have found that the index has a statistical difference. Although these results were similar, the specific reason is still unknown and further study is needed. Besides, according to the data of National Bureau of Statistics of the People’s Republic of China, the sex ratio of birth were 119.45, 117.94, 117.78, 117.70, 117.60 and 115.88 (female = 100) from 2009 to 2014, respectively, while the sex ratio aged 0–4 were 122.66, 119.15, 118.46 and 117.30 (female = 100) in the year 2009, 2011, 2012 and 2013, respectively. Few studies have focused on the relationship between the difference of prevalence in gender and the sex ratio of the crowd. Such studies may be able to reveal some of the pathogenesis from the perspective of sociology.

**Figure 1:**
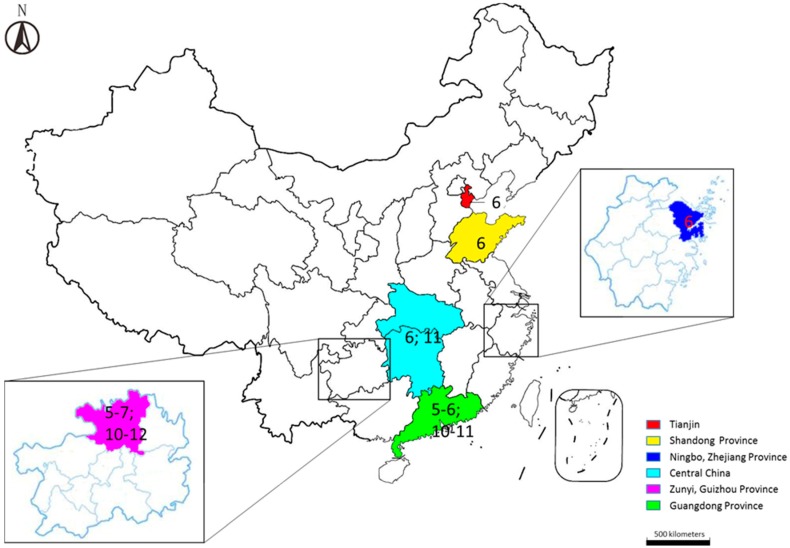
Locations of six regions in China. The numbers in the regions indicate the peak HFMD incidence month(s).

HFMD can occur year round, but the incidence has an apparent seasonal distribution in most regions of China [14,21,22,26,27,28,29,30,31,32,33] (Figure 1). Zhu *et al.* [26] suggested that the highest peak of HFMD cases was in April and the incidence remained high from April to August in Mainland China in 2008 and 2009. Ni *et al.* [28] found that the occurrence of HFMD reached a peak in June and that severe cases were diagnosed more frequently between May and August in Ningbo, while Zou *et al.* [27] observed two peaks of HFMD incidence in Guangzhou in April/May and September/October. Deng *et al.* [21] also showed two peaks in in Guangdong Province between 2008 and 2011, but both peaks occurred a month later than Zou’s report. Two peaks were also found in Zunyi from 2012 to 2014, according to Zhang *et al.* [22]. The first stronger wave was from May to July while the second weaker one was from October to December. Liu *et al.* [29] also found two obvious seasonal peaks of HFMD during the study period from 2011 to 2012. The first peak occurred from April to August with the highest peak in June and the second was from September to December with the highest in November. Moreover, Liu *et al.* [29] indicated that the seasonal distribution was significantly different between EV71 and CVA16. EV71 was the main causative agent during the first peak, while CVA16 predominated during the second peak and these two causative agents were significantly reduced in January and February, possibly because of the cold weather in these months. Tan *et al.* [30] indicated that the epidemic season of HFMD could extend from as early as May to as late as August in Tianjin from 2008 to 2013, while the peak usually occurred in June. Liu *et al.* [31] suggested that the incidence peak of HFMD appeared between April and August, while it was usually in June in Shandong Province from 2008 to 2012.

After an epidemiological study of HFMD from 2008 to 2012 in Mainland China, Xing *et al.* [14] indicated that the incidence peak of HFMD could be province-specific. At the national level, HFMD showed two peaks per year, including a strong peak in late spring and early summer and a weak peak in autumn. In southern China, the occurrence of HFMD had two peaks per year, in May and October, while it was different in northern China, where only one peak was experienced in summer, usually in June. With the increase of latitude, the semi-annual periodicity could be stronger. Accurate prediction of the incidence peak of HFMD is helpful to formulate a reasonable prevention and control strategy, and to reasonably allocate resources of disease prevention and control and medical resources. The prediction methods of the incidence peak of HFMD still needs to be further studied through comprehensive analysis of meteorological information, the incidence curve, and population information.

## 3. Etiology and Molecular Epidemiology

EV71 and CVA16 are the main causative agents of HFMD, while some other serotypes, including CVA4-10, CVA24, Coxsakievirus B2-5 (CVB2-5), and Echovirus 18 (ECHO18), can also lead to HFMD (Table 1) [30,34,35].

Based on the VP1 gene sequence, EV71 can be divided into three genotypes, A, B and C, and genotypes B and C can be further divided into several sub-genotypes, B1–5 and C1–5 [36]. Since the large range of HFMD caused by the EV71 outbreak with acute neurological complications in Mainland China in 2008, outbreaks have been reported every year with increasing morbidity and mortality. It is generally believed that severe and fatal cases along with neurological complications in large outbreaks are usually caused by sub-genotype C4a of EV71.

CVA16 was first isolated in South Africa in 1955, and has been causing HFMD in Asian and Pacific region for decades. The infection of CVA16 usually only cause mild cases, but it can sometime be severe and even lead to death [37]. CVA16 can also be divided into genotypes A, B1, and B2, and genotype B1 can be further divided into sub-genotypes B1a, B1b, and B1c [37,38].

**Table 1:** Genotypes of different HFMD pathogens isolated in various regions.

Pathogen	Region	Year	Genotype
Enterovirus 71	Jining, Shandong Province	2010	C1a
	Central China	2011–2012	45.2% (56/124) C1a
			54.8% (68/124) C1b
	Shijiazhuang, Hebei Province	2010–2012	C4a
Coxsackievirus A16	Linyi, Shandong Province	2009–2011	B1a
	Central China	2011–2012	31.25% (25/80) B1a
			68.75% (55/80) B1b
	Shijiazhuang, Hebei Province	2010–2012	B1
Coxsackievirus A6 *	Guangdong Province	2013	–
	Tianjin	2008–2013	–
	Fujian	2011–2013	–
Echovirus 30	Shandong Province	2010–2011	D2
Echovirus 25	Shandong Province	2010–2011	D6
Echovirus 6	Shandong Province	2010–2011	C6
Coxsackievirus B1	Shandong Province	2010–2011	D6
Coxsackievirus B3	Shijiazhuang, Hebei Province	2010–2012	E
Coxsackievirus B4	Shandong Province	2010–2011	D2
Coxsackievirus B10	Shijiazhuang, Hebei Province	2010–2012	C

* Because of the disunity of gene typing standards used in these studies, the genotypes are not listed here.

According to several studies [28,29,32,39,40,41,42,43], EV71 and CVA16 are still predominant in newly occurring HFMD cases. Du *et al.* [39] focused on the etiology of HFMD in Linyi, Shandong Province from 2009 to 2011 and suggested that the etiological spectrum and epidemic changes were in progress in Linyi. In the large epidemics in 2009 and 2010, EV71 was the predominant strain in the positive samples identified (63% and 82%, respectively), while CVA16 accounted for 16% and 15%, respectively. However, in 2011, EV71 only accounted for 11% of positive samples and CVA16 accounted for 51%. Moreover, the other enteroviruses rose to 38%, including CVA6 (6%), CVA12 (10%), Echovirus 30 (5%), CVA4 (6%), CVA2 (1%), CVB1 (3%), CVB4 (1%), and Echovirus 6 (1%). Furthermore, phylogenetic analysis showed that all CVA16 strains isolated in this study belonged to the B1a sub-genotype, which was consistent with other isolated strains from 1999 to 2008 in China. From May to October 2010, 579 HFMD patient samples were collected by Zhang *et al.* [40] for research on the prevalence of enterovirus in children with and without HFMD in Jining, Shandong Province. Among 579 cases, 440 were positive for Human Enterovirus (HEV), in addition, 43.7% were identified as EV71, while 18.0%, 5.7%, 3.8%, 1.0%, 0.5%, 0.4%, 0.4%, 0.2%, 0.2% 0.2% and 0.2% were identified as CVA16, CVA10, CVA6, CVA12, echovirus 9, echovirus 6, CVB6, CVA4, CVA14, echovirus 24, and echovirus 3, respectively. Moreover, the positive rates between mild and severe cases were significantly different for EV71 and CVA16 (39.7% *vs.* 55.0%, *p* = 0.001 and 20.0% *vs.* 11.9%, *p* = 0.024, respectively). In addition, according to the phylogenetic analysis, all the isolated EV71 strains belong to the C1a sub-genotype, which shares the most similarities with the strains isolated from Shandong Province. Liu *et al.* [29] analyzed 1844 HFMD cases hospitalized in Central China from 2011 to 2012. Molecular, epidemiological, and etiological studies indicated that 422 of 1884 cases were infected with CVA16, while 334 were infected with EV71, 38 were co-infected with EV71 and CVA16, and 34 were infected with other enteroviruses. Furthermore, 124 EV71 strains, 80 CVA16 strains, and nine other strains were isolated and analyzed among them. Phylogenetic analysis showed that 56 EV71 strains and 68 EV71 strains belonged to C1a and C1b sub-genotypes, respectively, while 25 CVA16 strains and 55 CVA16 strains belonged to B1a and B1b sub-genotypes, respectively. Though there was no difference between different causative agent-infected groups, the molecular study revealed that recombinant variants of CVA16 and EV71 were indicated and caused mild and severe cases.

In recent years, the proportion of EV71 and CVA16 infection in HFMD cases was reduced and many other enterovirus serotypes were detected and isolated in HFMD. Zhang *et al.* [22] carried out epidemic research of HFMD between 2012 and 2014 in Zunyi and suggested that in a total of 12,313 HFMD cases, 5750 cases were positive for viral detection, and among these cases 9%, 4.7%, and 46.7% were caused by EV71, CVA16, and other enteroviruses, respectively. Moreover, the overall infection rate of EV71 deceased from 33.3% in 2012 to 20% in 2014, and similar changes were observed in CVA16 infection, from 16% in 2012 to 13% in 2014. These data indicated that some other enterovirus causing HFMD had a trend toward a higher incidence rate among patients in Zunyi. However, a similar trend was also observed in Guangdong Province, according to Lu *et al.* [13]. In his research, 1,248,700 HFMD cases were collected from January of 2008 to August of 2013, with the help of a provincial web-based surveillance system established in 2008. From 2008 to 2012, EV71 and CVA16 were reported as the most predominant causative agents in Guangdong Province and EV71 represented about 40% of enterovirus-positive cases, except for 2009. While an obvious changing etiology was observed in 2013, CVA6 became the most isolated serotype in the new outbreak of HFMD in Guangdong Province. Moreover, phylogenetic analysis showed that the strains isolated in Guangdong Province during 2012 and 2013 epidemics revealed a close genetic relationship with the 2009/2010 Taiwan strains [44], 2010 French strains [45], and 2011 Japanese strains [46]. Since CVA6 had led to several outbreaks in many other countries [45,46,47,48,49], Lu *et al.* [13] provided evidence that surveillance of its circulation and further study were highly needed. Zhang *et al.* [35] analyzed some isolated strains of human enterovirus B-associated HFMD cases in Shandong Province from 2010 to 2011, indicating that the percentage of human enterovirus A-associated HFMD had dropped from 97% (2010) to 82.6% (2011), while the percentage of human enterovirus B-associated HFMD had increased from 3% (2010) to 17.4% (2011).

**Figure 2:**
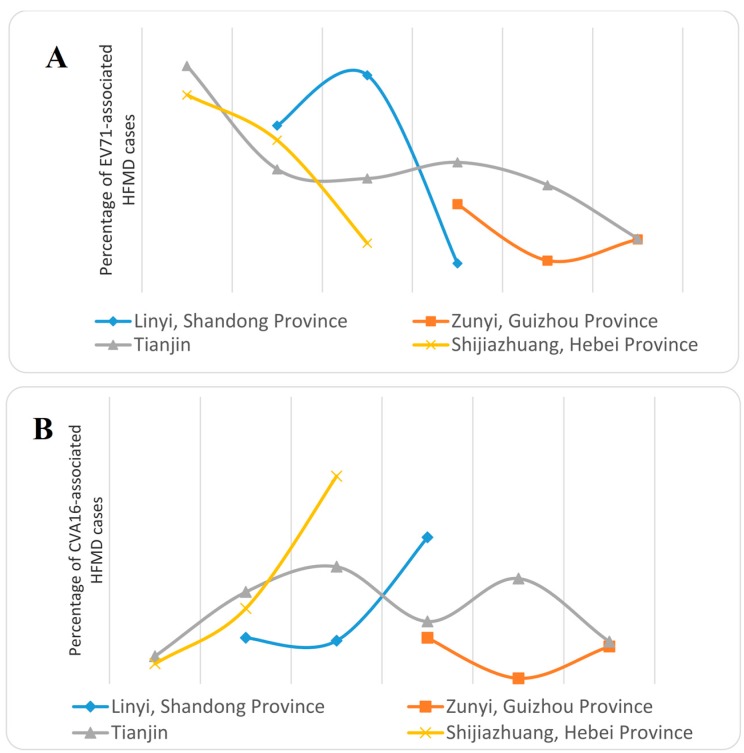

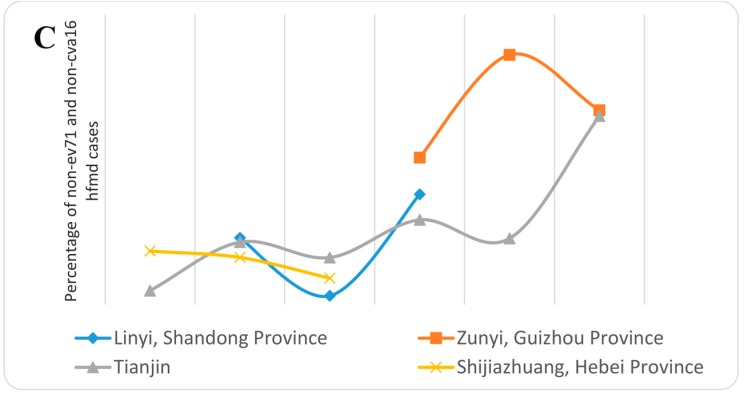
(**A**) Percentage of EV71-associated HFMD cases in different years; (**B**) Percentage of CVA16-associated HFMD cases in different years; (**C**) Percentage of non-EV71 and non-CVA116 HFMD cases in different years.

Moreover, ECHO30 was the most predominant of all the detected HEV-B serotypes. Others included ECHO25, CVA9, CVB1, CVB6, and ECHO6. In addition, molecular epidemiology and phylogenetic analysis based on the VP1 sequence of all these CVB1, CVB4, ECHO6, ECHO25, and ECHO30 isolated strains showed that the ECHO30 strains and ECHO25 strain belonged to novel sub-genotypes D2 and D6, respectively, while the ECHO6 strain, CVB1 strain, and CVB4 strain belonged to sub-lineage C6, subgroup D6 and sub-lineage D2, respectively. In addition, all these isolated strains occurred in genetic recombinations with other strains in specific regions, suggesting the importance of the prediction of potential emerging Human Enterovirus B(HEV-B)-associated HFMD outbreaks. In order to illustrate the molecular epidemiology of CVA6-caused HFMD, Chen *et al.* [50] analyzed 1340 non-EV71 and non-CVA16 HFMD cases in Fujian Province from 2011 to 2013. The result showed that of these cases, 375 cases were confirmed by viral isolation, and 182 cases were identified to be caused by CVA6. Moreover, CVA6-related HFMD accounted for 7.9%, 16.2%, and 39.6% enteroviruses-related HFMD cases in Fujian Province in 2011, 2012, and 2013, respectively. Molecular and phylogenetic analysis indicated that the CVA6 strains isolated from Fujian were different from the prototype strain, while similar to the domestic isolated strains. The results suggested that CVA6 strains were co-circulating and co-evolving with other enterovirus strains and had become one of the predominant strains of HFMD in Fujian Province. Coincidentally, according to the research carried out by Tan *et al.* [30] in Tianjin between 2008 and 2013, similar results were indicated. During 2008 to 2012, EV71 and CVA16 were the most predominant causative agents for HFMD, while in 2013, the major causative agent was CVA6. During 2008 to 2013, of 102,705 reported HFMD cases, 7892 patients had assays performed for EVs detection. The positive percentages for EV71 from 2008 to 2013 were 85.5%, 46.5%, 43.0%, 49.1%, 40.5%, and 20.3%, respectively, while for CVA16 they were 9.6%, 32.0%, 40.8%, 21.7%, 36.7%, and14.7%, respectively. In 2013, the positive percentage for detected CVA6 sharply increased, accounting for 54.3% of all the detected EVs cases. Analysis of VP1 sequence showed that all the isolated CVA6 strains could be divided into two clusters (cluster 1 and 2), which belonged to genotype D. Further phylogenetic analysis revealed that most of the CVA6 strains belonged to cluster 1, which showed a close relationship with the CVA6 strains prevalent in eastern and southern China in 2012 and 2013, showing a wide prevalence in Mainland China. The research by Tian *et al.* [34] in Shijiazhuang City, Hebei Province indicated that although EV71 and CVA16 were still the two predominant causative agents for HFMD, the trend seemed to be shifting: the infection of EV71 became weaker (74.56%, 57.47%, and 18.58% for 2010, 2011, and 2012, respectively) while CVA16 became stronger (6.99%, 26.28%, and 72.36% for 2010, 2011, and 2012, respectively), and CVA10 and CVB3 had also become important causative agents for HFMD, after the analysis of 4045 HFMD cases during 2010 to 2012. Phylogenetic analysis showed that the EV71 and CVA16 isolated strains belonged to C4a and B1 sub-genotypes, respectively, which were the same as the other strains circulating in Mainland China, while the CVB10 strains were divided into four different lineages which all belonged to genotype C and the CVB3 strains belonged to two lineages within genotype E. CVB10 and CVB3 strains also had the same origins as the strains isolated from other places in Mainland China. In conclusion, the prevalence of EV71 and CVA16 caused HFMD seems to become weaker, and many other causative agents including CVA6, ECHO30, and CVA10 have become more predominant in prevalence of HFMD. These results show the importance of research on these viruses (Figure 2).

At present, the cause of pathogenic spectrum changes is not clear. Perhaps, the following factors, virus variation, environmental condition, and susceptible population accumulation, play a role. An in depth study of the variation of the pathogenic spectrum is important for the prevention of diseases.

## 4. Influencing Factors

Several researchers found that the incidence of HFMD might be associated with some environmental factors [14,24,31,51,52,53,54,55,56]. After analysis of the epidemiological features of HFMD in Mainland China during 2008–2012, Xin *et al.* [14] indicated that evident geographical differences existed in the seasonal patterns of the incidence of HFMD between northern and southern China, and these differences were in weak associations with some climatic factors. By using geographical weighted regression models and working on 176,111 HFMD cases from 2912 counties in Mainland China, Hu *et al.* [52] pointed out that six climatic factors, including average-temperature (*AT*), average-minimum-temperature (*AT_min_*), average-maximum-temperature (*AT_max_*), average-temperature-difference (*AT_diff_*), average-relative-humidity (*ARH*), and monthly-precipitation (*MP*), all showed significant statistical relationships with the morbidity of HFMD, while the child population density (*CPD*) had a greater influence on morbidity than the climatic factors. The research carried out by Liu *et al.* [31] in Shandong Province, one of the most seriously HFMD affected regions in China, suggested that annual average temperature, annual average pressure, annual average relative humidity, annual average wind speed, and annual average sunshine hours had a significantly positive relationship with the incidence of HFMD, according to a best fitting Spatio-temporal interactive model. However, a study carried out by Wang *et al.* [51] provided evidence that vapor pressure and wind speed were significantly negatively related to the occurrence of HFMD in Shandong Province, while the average temperature and relative humidity showed positive results, using spatial panel data models. Similar results were observed by Li *et al.* [53]. The study used a negative binomial multivariable regression in the city of Guangzhou, located in southern China. Each 1 hPa rise in atmospheric pressure corresponded to a decrease in the number of cases by 6.80%. However, the increase in the number of cases corresponding to 1 °C rise in temperature, 1% rise in relative humidity, 1m/h in wind speed, and one-day addition in the number of windy days were 9.38%, 0.67% or 0.51%, 4.01% or 2.65%, 24.73%, and 25.87%, respectively. Through a spatial autologistic regression model, Bo *et al.* [54] identified that monthly average precipitation, monthly average temperature, and monthly average wind speed were statistically significant risk factors for the transmission of HFMD in Mainland China. Moreover, Bo’s study also suggested that the social-economic variables including the number of industrial enterprises above the designated size, the population density, and the proportion of the student population contributed to HFMD transmission. In addition to these risk factors, a researcher also identified ultraviolet radiation as a protective factor associated with the spread of HFMD. In addition, Xie *et al.* [24] demonstrated several risk factors associated with the transmission of HFMD, including having HFMD cases in the same class, having HFMD cases within the 20 nearest neighborhoods, exposure to public playgrounds, and finger sucking, and the odd ratios were 11.70 (95% confidence interval, 95% CI 1.26–109.40), 14.19 (95% CI 3.55–56.74), 6.03 (95 CI 2.84–12.80), and 2.13 (95% CI 1.05–4.32), respectively, from the research using univariate analysis followed by conditional logistic regression with attributable fraction computed. Moreover, washing hands with soap before meals seemed to be a protective factor (odd ratio, OR = 0.29, 95% CI 0.11–0.78), and these results indicated that good health education was beneficial to the prevention of HFMD transmission.

## 5. Detection and Surveillance

To achieve the goal of rapid and sensitive identification of causative agents from HFMD patients, several new method were developed [57,58]. Since CVA6 has become increasingly prevalent in many regions of China, a sensitive quantitatively real-time reverse-transcription polymerase chain reaction (RT-PCR) assay for rapid detection of CVA6 RNA was developed by Zhang *et al.* [57] By using bioinformatic technology, the conservation of the VP1 gene segment was analyzed and specific primers and probes were designed. The specificity was further confirmed and it could distinguish CVA6 from other similar viral infections, thus providing a reliable method for early diagnosis of CVA6 infection. Zhang *et al.* [58] developed a one-step, triplex, real-time RT-PCR assay in order to simultaneous detect EV71, CVA16, and pan-enteroviruses in a single tube as well as an internal amplification control. After detection for the continuous diluted viruses titrated by a 50% tissue culture infective dose (TCID_50_) assay, the results revealed that the lower detective limits were 0.02 TCID_50_/mL, 0.001 TCID_50_/mL, and 0.001–0.04 TCID_50_/mL for EV71, CVA16, and pan-enteroviruses, respectively. Moreover, the clinical application demonstrated high sensitivities for EV71, CVA16, and pan-enterovirus with a percentage of 98.7%, 100%, and 98.4%, respectively. This could help to identify causative agents from suspected cases.

In 2008, HFMD was listed as a C-class notifiable communicable disease and surveilled by the China Information System for Disease Control and Prevention. What is more, the researchers also established models for forecasting potential HFMD outbreaks in order to take some preventive measures for the control of HFMD [59,60]. Feng *et al.* [60] analyzed the etiology of HFMD cases from 2008 to 2012 in Zhengzhou, Henan Province using RT-PCR assay, and set up Seasonal Autoregressive Integrated Moving Average (SARIMA) models for the weekly number of HFMD, as well as EV71-associated and CVA16-associated HFMD, which were supposed to be two major causative agents for HFMD. The results revealed that the seasonal variation of HFMD pathogens can be related to climatic factors and the SARIMA system including climatic variables can effectively predicted the annual activity of HFMD epidemics. Similarly, Yu *et al.* [59] carried out a hybrid model consisting of the seasonal auto-regressive moving average (ARIMA) and nonlinear regressive neural network (NARNN) for predicting the potential incidence of HFMD from December 2012 to May 2013 in Shenzhen, Guangdong Province, by using retrospective observations from January 2008 to November 2012. The results showed that the model can effectively predict the occurrence trend of HFMD and help policy makers in making decisions and preventing epidemics.

## 6. Conclusions

The prevalence of HFMD in China is still an emergency situation. The infections of EV71 and CVA16 have caused enormous losses and new serotypes of enterovirus leading to HFMD have continuously emerged and gradually predominated. Though many studies have focused on EV71 and CVA16, studies on these new enterovirus serotypes are still few, so more studies are needed. Moreover, incessant genetic recombination makes the prevention of outbreaks a thing of the future. The relationship between meteorology and social factors and prevalence of HFMD can provide suggestions on protection of HFMD high-risk groups, the theoretical foundation for HFMD prevalence prediction, and references for policy making for disease prevention.
